# Deregulation of the miR-16-KRAS axis promotes colorectal cancer

**DOI:** 10.1038/srep37459

**Published:** 2016-11-18

**Authors:** Chaoying You, Hongwei Liang, Wu Sun, Jialu Li, Yanqing Liu, Qian Fan, Haiyang Zhang, Xin Yue, Jing Li, Xi Chen, Yi Ba

**Affiliations:** 1 Tianjin Medical University Cancer Institute and Hospital, National Clinical Research Center for Cancer, Key Laboratory of Cancer Prevention and Therapy, Huanhuxi Road, Tiyuanbei, Tianjin, 300060, China; 2 State Key Laboratory of Pharmaceutical Biotechnology, NJU Advanced Institute for Life Sciences (NAILS), Jiangsu Engineering Research Center for MicroRNA Biology and Biotechnology, School of Life Sciences, Nanjing University, 163 Xianlin Avenue, Nanjing, 210046, China; 3 Department of Gastroenterology, Tianjin First Center Hospital, 24 Fukang Road, Tianjin, 300192, China

## Abstract

KRAS plays a significant role in the etiology and progression of colorectal cancer (CRC), but the mechanism underlying this process has not been fully elucidated. In this study, we found that the KRAS protein levels were higher in CRC tissues than in the normal adjacent tissues, whereas its mRNA levels varied irregularly, suggesting that a post-transcriptional mechanism is involved in the regulation of KRAS. Then, we performed bioinformatic analyses to search for miRNAs that potentially target KRAS. We predicted and experimentally validated that miR-16 directly recognizes the 3′-UTR of the KRAS transcript and regulates KRAS expression. Furthermore, the *in vitro* results showed that the repression of KRAS by miR-16 suppressed the proliferation and invasion and induced the apoptosis of CRC cells, and the *in vivo* results revealed that miR-16 exerted a tumor-suppressive effect by negatively regulating KRAS in xenograft mice. Taken together, our findings provide evidence supporting the role of miR-16 as a tumor suppressor in CRC by targeting KRAS.

Colorectal cancer (CRC) is the third most commonly diagnosed cancer in males and ranks second in females, with 143,460 new cancer patients and 51,690 deaths estimated to occur in the United States in 2012^1^. The accumulation of genetic and non-genetic alterations as well as age, gender, high intake of fat, smoking, alcohol, obesity and a lack of physical exercise mediate the formation and progression of CRC^2^,^3^. At the genetic level, one of the most commonly activated signaling pathways in CRC is the KRAS signaling pathway, which has been associated with the occurrence and progression of intestinal neoplasms^4^. The KRAS (Kirsten rat sarcoma viral oncogene homolog) gene, located at 12p12.1, encodes a protein that is a member of the small GTPase superfamily. Abnormal activation of the KRAS protein alters the normal RAS/PI3K/AKT signaling and affects the biological behavior of cells, including cell proliferation, invasion and apoptosis^5^,^6^,^7^. Thus, KRAS belongs to a family of oncogenes that have the potential to cause normal cells to become cancerous. Abnormal expression and somatic activating mutations in KRAS are extensively found in various human cancers, including CRC, pancreatic cancer, gastric cancer, breast cancer and lung cancer^8^,^9^,^10^,^11^. Furthermore, activating oncogenic KRAS mutations are frequently associated with the resistance to chemotherapy and targeted therapies^12^,^13^,^14^,^15^,^16^. The search for small molecule inhibitors of activated KRAS with anticancer activity is intense.

MicroRNAs (miRNAs) are a class of non-coding, small, single-stranded, endogenous RNAs that play an important role in regulating gene expression at the post-transcriptional level^17^,^18^,^19^. Recent evidence has demonstrated that miRNAs can function as oncogenes or tumor suppressors via targeting cancer-related genes^20^. The abnormal expression of miRNAs plays a significant role in the development of CRC^21^, and some miRNAs directly regulate the proliferation, invasion and apoptosis of CRC cells^22^. For example, Ren *et al*. found that miR-206 is dramatically downregulated in CRC tissues and affects the proliferation, invasion and apoptosis of CRC cells by targeting FMNL2^23^. Wang *et al*. found that miR-320b functions as a tumor suppressor by targeting c-Myc in CRC cells and that the overexpression of miR-320b is closely correlated with a decrease of CRC cell growth *in vitro* and *in vivo*^24^. Li *et al*. found that miR-766 is dramatically upregulated in CRC tissues and cells and promotes cell proliferation by targeting SOX6^25^. However, the molecular mechanism underlying the contribution of miRNAs to the development and progression of CRC remains to be elucidated.

Although KRAS and miRNAs are well known to be associated with carcinogenesis in human CRC, the molecular mechanism underlying their widespread dysregulation is not fully understood. While some miRNAs have been reported to target KRAS^26^,^27^,^28^, the detailed roles of miRNAs and KRAS and their interactions in the initiation and progression of CRC remain to be fully elucidated. The aim of this study is to evaluate the association of miRNAs with KRAS and identify new miRNAs that can act on KRAS. Here, we found that KRAS is directly regulated by miR-16 in CRC cells. Furthermore, we showed that miR-16 suppresses the proliferation and invasion and induces the apoptosis of CRC cells by inhibiting KRAS expression. In addition, we constructed tumor xenografts in mice and showed that miR-16 inhibits tumor growth, partially by negatively regulating KRAS expression.

## Materials and Methods

### Human tissues

Sixteen CRC patients who underwent surgical resection at the Tianjin Medical University Cancer Institute and Hospital (Tianjin, China) were enrolled in this study. Paired CRC tissues and adjacent non-tumor tissues were obtained from the patients. Both the tumor and the non-tumor tissues were sent for histological analysis for diagnostic confirmation. The pathological types of all tumors were identified as adenocarcinoma. All protocols concerning the use of patient samples in this study were approved by the ethics committee of Tianjin Medical University Cancer Institute and Hospital, and all patients signed informed consent for the collection and use of their tissues for this study. The methods were carried out in accordance with the approved guidelines by Tianjin Medical University Cancer Institute and Hospital. The clinical data from the patients are listed in Table 1. Tissue fragments were promptly frozen in liquid nitrogen at the time of surgery and stored at −80 °C.

### Cell culture

The human CRC cell lines SW480, HT-29 and Caco2 cells were purchased from the Shanghai Institute of Biochemistry and Cell Biology, Chinese Academy of Sciences (Shanghai, China). SW480 and HT-29 cells were cultured in RPMI-1640 medium (Gibco, CA, USA) supplemented with 10% fetal bovine serum (FBS, Gibco), and Caco2 cells were cultured in RPMI-DMEM medium (Gibco) supplemented with 10% FBS. All cells were cultured in a humidified incubator at 37 °C containing 5% CO_2_.

### RNA isolation and quantitative RT-PCR

Total RNA was extracted from the cultured cells and human tissue specimens using Trizol reagent (Invitrogen, CA, USA) according to the manufacturer’s instructions. Assays to quantify miRNAs were processed using TaqMan miRNA probes (Applied Biosystems, CA, USA) according to the manufacturer’s instructions. Briefly, 1 μg of total RNA was reverse-transcribed to cDNA using a stem-loop RT primer (Applied Biosystems) and AMV reverse transcriptase (TaKaRa, Dalian, China). The reaction conditions were as follows: 16 °C for 30 min, 42 °C for 30 min and 85 °C for 5 min. Quantitative real-time PCR was processed using a TaqMan PCR kit on an Applied Biosystems 7300 Sequence Detection System (Applied Biosystems). The reaction conditions were 95 °C for 5 min, followed by 40 cycles of 95 °C for 15 s and 60 °C for 1 min. All the reactions were processed in triplicate. After all reactions were completed, the cycle threshold (C_T_) data were gathered using fixed threshold settings, and the mean C_T_ values were determined from triplicate PCRs. U6 snRNA was used as an internal control, and the amount of miRNA normalized to the U6 level was calculated using the formula 2^−ΔΔCT^, in which ΔΔC_T_ = (C_TmiRNA_ − C_TU6_)_tumor_ − (C_TmiRNA_ − C_TU6_)_control_.

To quantify the KRAS mRNA, 1 μg of total RNA was reverse-transcribed to cDNA using oligodT (TaKaRa) and AMV reverse transcriptase (TaKaRa). The reaction conditions were: 16 °C for 30 min, 42 °C for 30 min and 85 °C for 5 min. Real-time PCR was then processed using the RT product, SYBR Green dye (Invitrogen) and specific primers for KRAS and GAPDH. The relative amount of KRAS mRNA was normalized to GAPDH. The sequences of the primers were as follows: KRAS (sense): 5′-GACTCTGAAGATGTACCTATGGTCCTA-3′; KRAS (antisense): 5′-CATCATCAACACCCTGTCTTGTC-3′; GAPDH (sense): 5′-GATATTGTTGCCATCAATGAC-3′; and GAPDH (antisense): 5′-TTGATTTTGGAGGGATCTCG-3′. The reactions were incubated at 95 °C for 5 min, followed by 40 cycles of 95 °C for 30 s, 60 °C for 30 s and 72 °C for 30 s.

### Overexpression or knockdown of miR-16

Overexpression of miR-16 was achieved by transfecting cells with pre-miR-16 (a synthetic RNA oligonucleotide mimicking the miR-16 precursor). Knockdown was achieved by transfecting a miRNA inhibitor (a chemically modified single-stranded antisense oligonucleotide designed to specifically sequester the mature miRNA). Synthetic pre-miR-16, anti-miR-16 and scrambled negative control RNA (pre-miR-control and anti-miR-control) were purchased from GenePharma (Shanghai, China). SW480, HT-29 and Caco2 cells were seeded in 6-well plates. On the following day, the cells were transfected using Lipofectamine 2000 (Invitrogen) when the cells were approximately 70% confluent. In each well, 100 pmol of pre-miR-16, pre-miR-control, anti-miR-control and anti-miR-16 were used. After 4–6 h, the media were changed to RPMI-1640 or RPMI-DMEM medium supplemented with 2% FBS. The cells were harvested at 24 or 48 h after transfection for the isolation of total RNA or protein, respectively.

### Plasmid construction and siRNA interference assay

A mammalian expression plasmid designed to specifically express the open reading frame (ORF) of human KRAS without the miR-16-responsive 3′-UTR was purchased from GeneCopoeia (Germantown, MD, USA). An empty plasmid served as a negative control. Four siRNA sequences targeting different sites of human KRAS cDNA (si-KRAS) were synthesized by GenePharm. siRNA sequences were designed according to previous reports^29^,^30^. A scrambled siRNA (GenePharma) was used as a negative control. The siRNA sequences were as follows: si-KRAS#1: 5′-GGUGACUUAGGUUCUAGAUTT-3′; si-KRAS#2: 5′-GGAAGCAAGUAGUAAUUGATT-3′; si-KRAS#3: 5′-CGAAUAUGAUCCAACAAUATT-3′; and si-KRAS#4: 5′- UAAGGACUCUGAAGAUGUATT-3′. The KRAS overexpression plasmid or siRNA was transfected into SW480 cells using Lipofectamine 2000 (Invitrogen) according to the manufacturer’s instructions. Total RNA or protein was isolated 24 or 48 h post-transfection. The KRAS mRNA and protein expression levels were assessed by quantitative RT-PCR and Western blot, respectively.

### Luciferase reporter assay

The entire 3′-UTR of human KRAS was amplified via PCR with human genomic DNA as a template. Then, the PCR products were inserted into the pMIR-REPORT plasmid (Ambion, USA), and successful insertion was confirmed by DNA sequencing. To evaluate the binding specificity, the sequences that interacted with the seed region of miR-16 were mutated (from TGCTGCT to ACGACGA), and the mutant KRAS 3′-UTR was inserted into an equivalent luciferase reporter. For luciferase reporter assays, SW480 or Caco2 cells were seeded in 24-well plates and co-transfected with 1 μg of the firefly luciferase reporter plasmid, 1 μg of the β-galactosidase (β-gal) expression plasmid (Ambion), and equal amounts (100 pmol) of pre-miR-control, pre-miR-16 or anti-miR-control, anti-miR-16 using Lipofectamine 2000 (Invitrogen). A β-gal plasmid was used as a transfection efficiency control. The cells were harvested 24 h post-transfection and were assayed with a luciferase assay kit (Promega, Madison, WI, USA).

### Protein isolation and Western blot

Cells or tissues were lysed in RIPA lysis buffer (Beyotime, China) containing a protease inhibitor cocktail (Thermo Scientific 78440) for 30 min on ice and were then centrifuged at 12,000 × g at 4 °C for 10 min. The supernatant was collected, and the protein concentration was calculated using a BCA protein assay kit (Thermo Scientific, Rockford, USA). The protein levels were analyzed via Western blotting using the corresponding antibodies and normalized by probing the same blots using a GAPDH antibody. The antibodies against KRAS and GAPDH were purchased from Santa Cruz Biotechnology (sc-30 and sc-365062, Santa Cruz, CA, USA). The parent unmodified Western blot images without cropping are shown in Supplementary Fig. 4.

### Cell proliferation assay

To assess cell proliferation, SW480 and Caco2 cells were seeded in triplicate at a density of 1 × 10^4^ cells per well in 96-well plates and then incubated overnight in 100 μl RPMI-1640 or RPMI-DMEM medium supplemented with 10% FBS. The cell proliferation index was measured using the Cell Counting Kit-8 (CK04-500, Dojindo, Japan) at 12, 24, 36, 48 and 60 h after transfection according to the manufacturer’s instructions. All experiments were performed in triplicate.

### Cell invasion assay

The invasion ability of SW480 and Caco2 cells was tested using Matrigel Invasion Chambers (BD Biosciences, MA, USA) with inserts containing an 8-μm pore-size membrane. The membranes on the bottom of the upper compartment of the Transwells were coated with a thin layer of Matrigel. The cells were harvested 24 h after transfection, suspended in FBS-free RPMI-1640 or RPMI-DMEM culture medium and added to the upper chamber (4 × 10^4^ cells per well). At the same time, 0.6 ml of RPMI-1640 or RPMI-DMEM with 20% FBS was added to the lower compartment. The cells were allowed to invade for 24 h in a 5% CO_2_ atmosphere that was saturated with H_2_O at 37 °C. After incubation, cells that entered the lower surface of the filter membrane were fixed with 4% paraformaldehyde for 20 min at room temperature, washed 2 times with 1 × PBS and stained with 0.5% crystal violet solution for 15 min at room temperature. The cells remaining in the upper surface of the filter membrane (non-migrant) were scraped out gently with a cotton swab. The lower surfaces (with cells that had migrated) were imaged using a photomicroscope (10 ×  fields per chamber) (BX51, Olympus, Japan). All experiments were performed in triplicate.

### Apoptosis assays

The apoptosis of SW480 and Caco2 cells was tested using an Annexin V-FITC/propidium iodide (PI) staining assay. The SW480 cells were cultured in 12-well plates and transfected with pre-miR-16, KRAS siRNA or the KRAS overexpression plasmid and the Caco2 cells transfected with anti-miR-16 to induce apoptosis. Pre-miR-control, anti-miR-control, control siRNA and a control plasmid served as negative controls. The cells were cultured for 48 h in FBS-free RPMI-1640 medium or RPMI-DMEM, and then the attached and floating cells were harvested. Flow cytometric analysis of the apoptotic cells was performed using an Annexin V-FITC/PI staining kit (BD Biosciences, CA, USA). After washing with cold PBS twice, the cells were resuspended in binding buffer (100 mM HEPES, pH 7.4; 100 mM NaCl; 25 mM CaCl_2_), followed by staining with Annexin V-FITC/PI at room temperature in the dark for 15 min. The apoptotic cells were then evaluated by gating the PI- and Annexin V-positive cells with a fluorescence-activated cell-sorting (FACS) flow cytometer (BD Biosciences, San Jose, USA). All experiments were performed in triplicate.

### Establishment of tumor xenografts in mice

Five-week-old male C57BL/6J nude mice were purchased from the Model Animal Research Center of Nanjing University (Nanjing, China) and housed under specific pathogen-free conditions at Nanjing University. SW480 cells were infected with the miR-16 overexpression lentivirus alone, transfected with the KRAS overexpression plasmid alone, or co-transfected with the miR-16 overexpression lentivirus and the KRAS overexpression plasmid. After infection and transfection, SW480 cells were subcutaneously injected into C57BL/6J nude mice (2 × 10^6^ cells per mouse, 6 mice per group). Mice injected with untreated SW480 cells served as controls. The mice were sacrificed one month later. The tumors were separated from the animals, and the tumor weights were measured. Parts of the tissues were used for protein and total RNA extraction, and the remaining tissues were fixed in 4% paraformaldehyde for 24 h and then processed for Hematoxylin and eosin (H&E) staining and immunohistochemical staining for KRAS and Ki-67. All experiments were approved by the Institutional Review Board of Nanjing University (Nanjing, China) and performed in accordance with the guidelines of the National Institutes.

### Statistical analysis

All the Western blot images are representative of at least three independent experiments. The quantitative RT-PCR assays, luciferase reporter assays, proliferation assays, invasion assays and apoptosis assays were performed in triplicate, and each experiment was repeated several times. The results are shown as the means ± SD of at least three independent experiments. The numerical data were statistically analyzed by two-tailed Student’s t-test. Bivariate correlation between two independent variables was calculated by Spearman’s rank correlation co-efficient. All the differences were considered statistically significant at p < 0.05.

## Results

### Upregulation of the KRAS protein, but not mRNA, in CRC tissues

**Figure 1:**
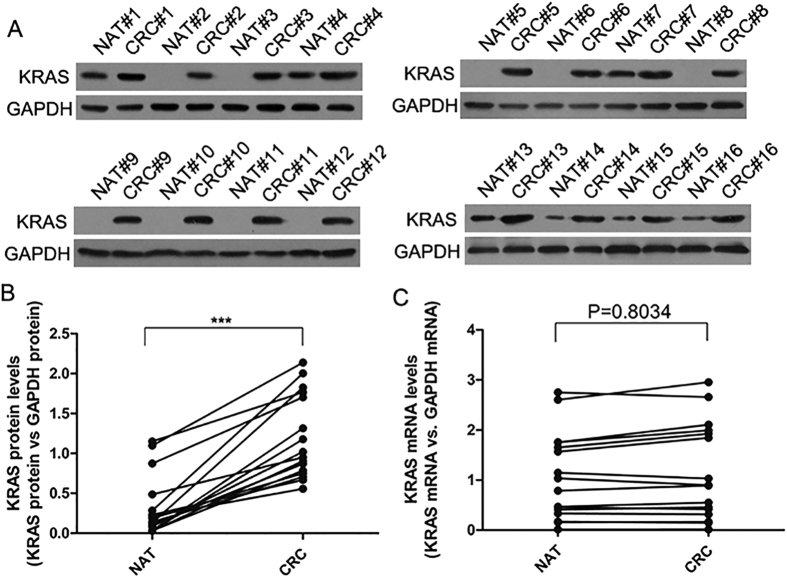
The expression of KRAS in human CRC tissues. (**A** and **B**) Western blot analysis of the KRAS protein levels in 16 pairs of CRC and NAT samples. GAPDH was used as a loading control. A: representative image; B: Quantitative analysis (KRAS protein vs. GAPDH protein). (**C**) Quantitative RT-PCR analysis of the KRAS mRNA levels (KRAS mRNA vs. GAPDH mRNA) in the same 16 pairs of CRC and NAT samples. (mean ± S.D; ***p < 0.001).

We first determined the expression patterns of the KRAS protein in human CRC tissues. We found that the KRAS protein expression levels were significantly higher in the CRC tissues than in the normal adjacent tissue (NAT) samples (Fig. 1A and B). The mean expression levels of KRAS protein in the CRC and NAT samples are shown in Supplementary Fig. 1A. However, although the KRAS protein was consistently upregulated in the CRC samples, KRAS mRNA levels appeared to be irregular between the tumor and paired non-tumor samples (Fig. 1C). The mean expression levels of KRAS mRNA in the CRC and NAT samples are shown in Supplementary Fig. 1B. This disparity between the KRAS protein and mRNA expression in the CRC tissues suggests that a post-transcriptional mechanism is involved in the regulation of KRAS. However, it is noted that a very large difference was observed in expression of the KRAS in different samples (e.g., NAT#2 vs. NAT#4). Such inconsistency may be due to different age, gender and pathological stages of the CRC patients.

### Prediction of conserved miR-16 binding sites within the 3′-UTR of KRAS

**Figure 2:**
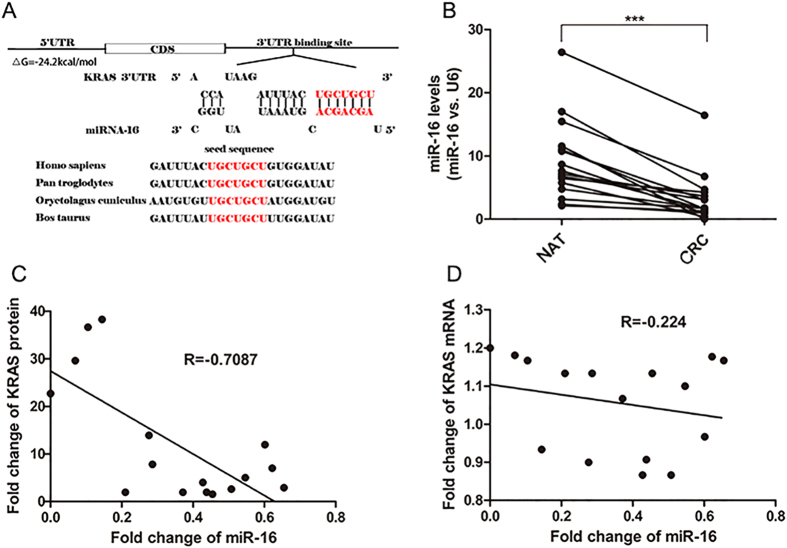
Prediction of KRAS as the target of miR-16. (**A**) Schematic description of the hypothetical duplexes formed by the interactions between the binding site in the KRAS 3′-UTR (top) and miR-16 (bottom). The seed region of miR-16 and the seed-recognizing site in the KRAS 3′-UTR are indicated in red, and all nucleotides in seed-recognizing site are completely conserved in several species. The predicted free energy value of the hybrid is indicated. (**B**) Quantitative RT-PCR analysis of the miR-16 expression levels (miR-16 vs. U6) in the same 16 pairs of CRC and NAT samples. (**C**) Pearson’s correlation scatter plot of the fold change of miR-16 and KRAS protein in CRC samples. (**D**) Pearson’s correlation scatter plot of the fold change of miR-16 and KRAS mRNA in CRC samples. (mean ± S.D.; ***p < 0.001).

One important mode of post-transcriptional regulation is the repression of mRNA transcripts through miRNAs. Three algorithms (TargetScan^31^, miRanda^32^ and PicTar^33^) were used in combination to identify potential miRNAs that target KRAS. All three algorithms predicted miR-16 as a candidate miRNA that targets KRAS. The predicted interaction between miR-16 and KRAS mRNA is illustrated in Fig. 2A. One potential binding site in the 3′-UTR of KRAS mRNA was observed. There was perfect base-pairing between the seed region (the core sequence that encompasses the first 2–8 bases of the mature miRNA) and the cognate target. The minimum free energy value of the hybridization between miR-16 and KRAS mRNA was −24.2 kcal/mol, which is well within the range of genuine miRNA target pairs^34^,^35^. Furthermore, the miR-16 binding sequence in the KRAS 3′-UTR was highly conserved across species.

We then investigated whether the expression levels of miR-16 were inversely correlated with the levels of the KRAS protein in CRC tissues. We measured the miR-16 levels in the same paired CRC and NAT samples and found that miR-16 was significantly decreased in CRC samples (Fig. 2B), which is consistent with the notion that the levels of miRNAs are opposite to that of their targets. The mean expression levels of miR-16 in the CRC and NAT samples are shown in Supplementary Fig. 1C. The inverse correction between miR-16 and KRAS protein levels (Fig. 2C) and the disparity between the miR-16 and KRAS mRNA levels (Fig. 2D) were further illustrated using Pearson’s correction scatter plots.

### Validation of KRAS as a direct target of miR-16

**Figure 3:**
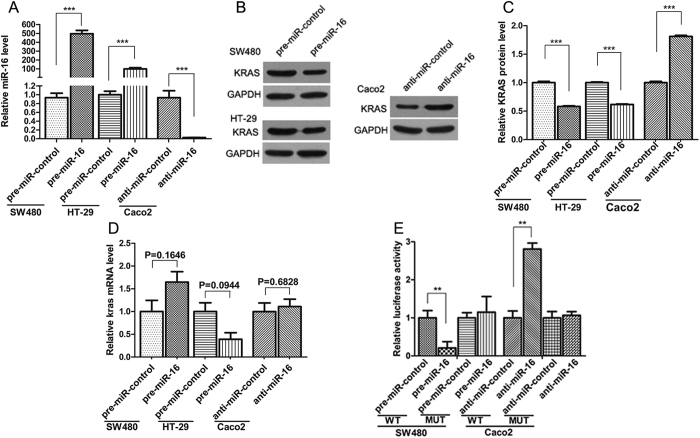
KRAS is a direct target of miR-16. (**A**) Quantitative RT-PCR analysis of the miR-16 expression levels in SW480 and HT-29 cells that were transfected with pre-miR-16 or pre-miR-control and in Caco2 cells that were transfected with anti-miR-16 or anti-miR-control for 24 h. (**B** and **C**) Western blot analysis of the KRAS protein levels in SW480 and HT-29 cells that were transfected with pre-miR-16 or pre-miR-control and in Caco2 cells that were transfected with anti-miR-16 or anti-miR-control for 48 h. B: representative image; C: quantitative analysis. (**D**) Quantitative RT-PCR analysis of the KRAS mRNA expression levels in SW480 and HT-29 cells that were transfected with pre-miR-16 or pre-miR-control and in Caco2 cells that were transfected with anti-miR-16 or anti-miR-control for 24 h. (**E**) Direct recognition of the KRAS 3′-UTR by miR-16. Firefly luciferase reporters containing either the wild-type (WT) or mutant (MUT) form of the human KRAS 3′-UTR were co-transfected into SW480 cells along with pre-miR-16 or pre-miR-control, or into Caco2 cells along with anti-miR-16 or anti-miR-control. At 24 h post-transfection, the cells were assayed using a luciferase assay kit. Firefly luciferase values were normalized to β-gal activity and plotted as relative luciferase activity. For comparison, the luciferase activity in pre-miR-control-transfected and anti-miR-control-transfected cells was set as 1. (mean ± S.D.; **p < 0.01; ***p < 0.001).

The correlation between miR-16 and KRAS was examined by evaluating the levels of KRAS in human CRC cell lines after the overexpression or knockdown of miR-16. Firstly, we examined the inherent expression levels of miR-16 in three CRC cell lines, SW480, HT-29 and Caco2. The result indicated that miR-16 levels were significantly lower in SW480 and HT-29 cells than in Caco2 cells. Thus, we selected SW480 and HT-29 cells to perform the gain of function experiments and selected Caco2 cells to perform the loss of function experiments for miR-16. MiR-16 overexpression was achieved by transfecting SW480 and HT-29 cells with pre-miR-16, whereas miR-16 knockdown was achieved by transfecting Caco2 cells with anti-miR-16. The efficient overexpression of miR-16 in SW480 and HT-29 cells and knockdown of miR-16 in Caco2 cells was shown in Fig. 3A. As anticipated, the overexpression of miR-16 dramatically reduced the KRAS protein levels in SW480 and HT29 cells, whereas the knockdown of miR-16 significantly increased the KRAS protein levels in Caco2 cells (Fig. 3B and C). We also determined the level at which miR-16 regulates KRAS expression. By measuring the expression of KRAS mRNA after transfecting CRC cells with pre-miR-16 or anti-miR-16, we found that miR-16 did not significantly affect the mRNA levels of KRAS (Fig. 3D). These results demonstrate that miR-16 specifically regulates KRAS expression at the post-transcriptional level.

To determine whether the negative regulatory effect of miR-16 on KRAS expression was mediated by the binding of miR-16 to the predicted target sites in the 3′-UTR of the KRAS mRNA, the KRAS 3′-UTR containing the predicted miR-16 binding site was inserted the downstream of the firefly luciferase gene in a reporter plasmid. The resulting plasmid was transfected into SW480 cells along with either pre-miR-16 or pre-miR-control, or into Caco2 cells along with either anti-miR-16 or anti-miR-control. As expected, the luciferase activity was remarkably reduced in SW480 cells transfected with pre-miR-16 compared to cells transfected with pre-miR-control, whereas the luciferase activity was significantly increased in Caco2 cells transfected with anti-miR-16 compared to cells transfected with anti-miR-control (Fig. 3E). Furthermore, we introduced point mutations into the corresponding complementary sites in the 3′-UTR of KRAS to eliminate the predicted miR-16 binding site. The mutated luciferase reporter was unaffected by the overexpression or knockdown of miR-16 (Fig. 3E). This result suggests that the putative miR-16 binding site of KRAS strongly contributes to the miRNA-mRNA interaction.

### miR-16 inhibits cell proliferation and invasion and promotes cell apoptosis by targeting KRAS

**Figure 4:**
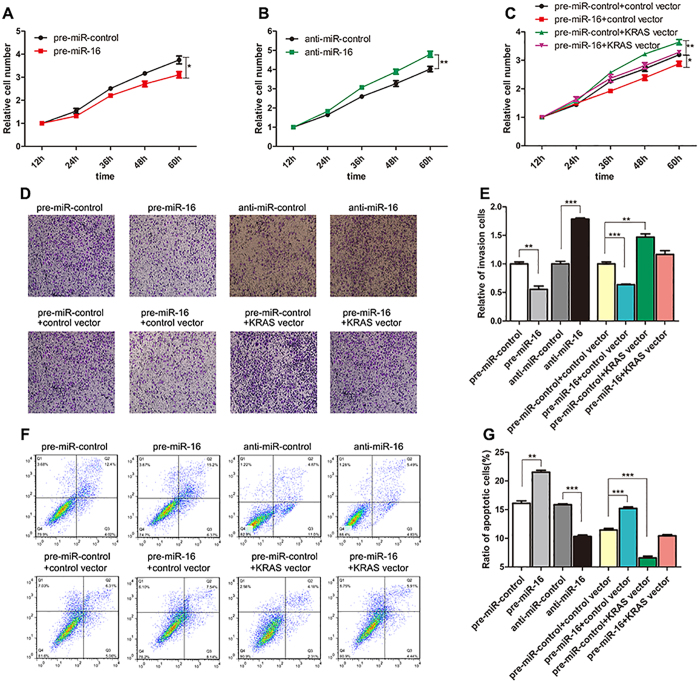
Effect of miR-16 and KRAS on the proliferation, invasion and apoptosis of CRC cells. (**A**) Cell proliferation assays were performed 12, 24, 36, 48 and 60 h after the transfection of SW480 cells with pre-miR-16 or pre-miR-control. (**B**) Cell proliferation assays were performed 12, 24, 36, 48 and 60 h after the transfection of Caco2 cells with anti-miR-16 or anti-miR-control. (**C**) Cell proliferation assays were performed 12, 24, 36, 48 and 60 h after the transfection of SW480 cells with pre-miR-control plus a control plasmid, pre-miR-control plus a KRAS overexpression plasmid, pre-miR-16 plus a control plasmid, or pre-miR-16 plus a KRAS overexpression plasmid. (**D** and **E**) Transwell analysis of SW480 cells transfected with pre-miR-16 or pre-miR-control, or with pre-miR-control plus a control plasmid, pre-miR-control plus a KRAS overexpression plasmid, pre-miR-16 plus a control plasmid, or pre-miR-16 plus a KRAS overexpression plasmid. At the same time, Caco2 cells were transfected with anti-miR-16 or anti-miR-control and then subjected to Transwell analysis. D: representative image; E: quantitative analysis. (**F** and **G**) An apoptosis assay was performed 48 h after the transfection of SW480 cells with pre-miR-16 or pre-miR-control, or with pre-miR-control plus a control plasmid, pre-miR-control plus a KRAS overexpression plasmid, pre-miR-16 plus a control plasmid, or pre-miR-16 plus a KRAS overexpression plasmid. At the same time, Caco2 cells were transfected with anti-miR-16 or anti-miR-control and then subjected to apoptosis analysis. Cell apoptosis profiles were analyzed by flow cytometry. F: representative image; G: quantitative analysis. (mean ± S.D.; *p < 0.05; **p < 0.01; ***p < 0.001).

We next analyzed the biological consequences of the repression of KRAS expression caused by miR-16 in CRC cells. Firstly, SW480 and Caco2 cells were transfected with pre-miR-control or anti-miR-16, respectively, and then the changes in cell proliferation were analyzed. Compared with the control cells, SW480 cells transfected with pre-miR-16 showed a significant reduction of the cell proliferation rate, whereas anti-miR-16 showed an opposite effect on cell proliferation in Caco2 cells (Fig. 4A and B). Then, we examined whether the overexpression or knockdown of KRAS would impact the proliferation of SW480 cells. To knockdown KRAS, four siRNA sequences targeting different sites of KRAS cDNA were synthesized, and the sequence with the best interfering effect (si-KRAS#2) was selected. To overexpress KRAS, an expression plasmid designed to specifically express the full-length KRAS ORF without the miR-16-responsive 3′-UTR was constructed. As expected, the cells transfected with KRAS siRNA showed a reduction of KRAS mRNA and protein levels, whereas the cells transfected with a KRAS overexpression plasmid showed increased KRAS mRNA and protein levels (Supplementary Fig. 2A–C). Consequently, cells transfected with KRAS siRNA proliferated at a significantly lower rate than the control siRNA-transfected cells (Supplementary Fig. 2D), whereas the cell proliferation rate was increased in the cells transfected with the KRAS overexpression plasmid compared to the cells transfected with an empty control plasmid (Supplementary Fig. 2E). Thus, miR-16 and KRAS have opposite effects on cell proliferation. Subsequently, we investigated whether the overexpression of miR-16-resistant KRAS (KRAS ORF) was sufficient to rescue the suppression of KRAS by miR-16 and attenuated the anti-proliferative effect of miR-16 in CRC cells. Cells transfected with pre-miR-16 and the KRAS overexpression plasmid showed significantly higher proliferation rates than cells transfected with pre-miR-16 alone (Fig. 4C), suggesting that the overexpression of KRAS rescued the miR-16-mediated downregulation of the proliferation rates of SW480 cells. Taken together, the results indicate that miR-16 can inhibit cell proliferation by silencing KRAS.

Furthermore, we assessed the effect of miR-16 and KRAS on the invasion ability of SW480 and Caco2 cells. The invasion assays indicated that the invasion rate of SW480 cells transfected with pre-miR-16 was significantly decreased compared to control cells, whereas anti-miR-16 increased the invasion ability of Caco2 cells (Fig. 4D and E). Transfection of KRAS siRNA dramatically reduced the number of SW480 cells that pass through the Transwell membrane, whereas transfection of the KRAS overexpression plasmid increased the invasion rate (Supplementary Fig. 2F and G). Thus, the inhibition of cell invasion by KRAS knockdown was similar to that elicited by miR-16 overexpression, further indicating that miR-16 and KRAS have opposite effects on cell invasion. Moreover, the overexpression of miR-16-resistant KRAS remarkably attenuated the anti-invasion effect of miR-16 when SW480 cells were co-transfected with pre-miR-16 and the KRAS overexpression plasmid (Fig. 4D and E). In summary, these results suggest that miR-16 may suppress cell invasion by silencing KRAS.

We finally investigated apoptosis in SW480 and Caco2 cells via flow cytometric analysis. In SW480 cells with enhanced miR-16 expression, the percentage of apoptotic cells was significantly higher than in the control cells, whereas anti-miR-16 had the opposite effect on cell apoptosis in Caco2 cells (Fig. 4F and G). In addition, KRAS interference remarkably increased the percentage of apoptotic cells, whereas KRAS overexpression decreased cell apoptosis (Supplementary Fig. 2H and I). Moreover, when SW480 cells were co-transfected with pre-miR-16 and the KRAS overexpression plasmid, KRAS dramatically attenuated the miR-16-induced apoptosis effect (Fig. 4E and F), suggesting that KRAS might reverse the pro-apoptosis effect of miR-16. The results indicate that miR-16 may modulate cell apoptosis by downregulating KRAS.

### The effect of miR-16 and KRAS on the growth of CRC cells *in vivo*

**Figure 5:**
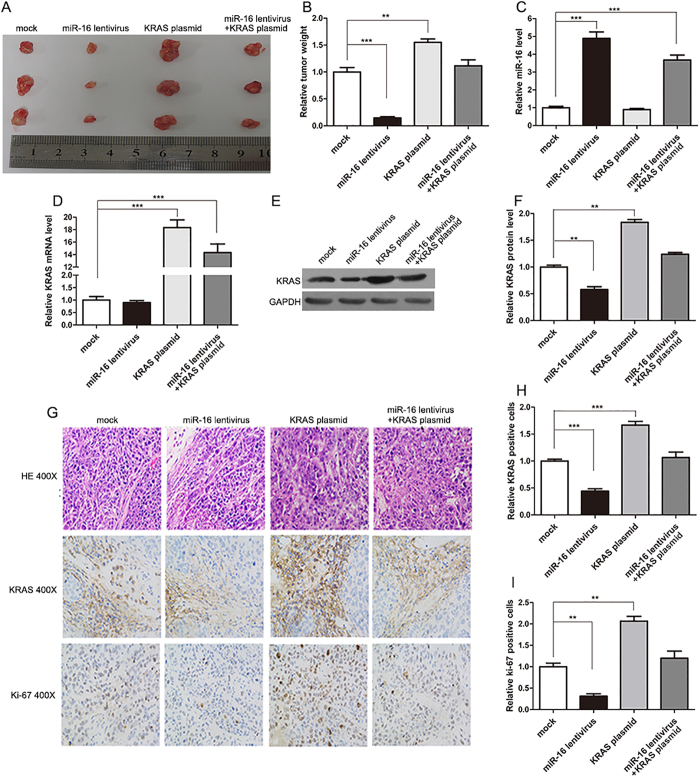
Effects of miR-16 and KRAS on the growth of CRC xenografts in mice. SW480 cells were untreated (mock), infected with a lentivirus to overexpress miR-16, transfected with a plasmid to overexpress KRAS, or co-transfected with a lentivirus to overexpress miR-16 and a plasmid to overexpress KRAS. After the different treatments, SW480 cells (2 × 10^6^ cells per mouse) were then implanted subcutaneously into 5-week-old male C57BL/6J nude mice (6 mice per group), and tumor growth was evaluated at day 30 after cell implantation. (**A**) Representative images of the tumors from the implanted mice. (**B**) Quantitative analysis of the tumor weights. **(C)** Quantitative RT-PCR analysis of the miR-16 levels in tumors from the implanted mice. (**D**) Quantitative RT-PCR analysis of the KRAS mRNA levels in tumors from the implanted mice. (**E** and **F**) Western blotting analysis of the KRAS protein levels in tumors from the implanted mice. (**E**) representative image; (**F**) quantitative analysis. (**G–I**) H&E-stained sections and immunohistochemical staining for KRAS and Ki-67 in tumors from the implanted mice. (**G**) representative image; (**H** and **I**) quantitative analysis. (mean ± S.D.; **p < 0.01; ***p < 0.001).

We next evaluated the effects of miR-16 and KRAS on the growth of CRC xenografts in mice. The use of viral constructs provides the possibility to rapidly produce high intracellular levels of mature miRNA via the endogenous miRNA processing pathway. To generate a viral expression construct, a 300-bp fragment containing the genomic sequences of miR-16 was obtained by PCR amplification of human genomic DNA and cloned into a lentiviral expressing vector. SW480 cells were infected with the miR-16 overexpression lentivirus. The expression of mature miR-16 was observed to be 8-10-fold higher after infection (Supplementary Fig. 3A), and KRAS protein expression was consequently inhibited (Supplementary Fig. 3B and C). Subsequently, SW480 cells were infected with the miR-16-overexpressing lentivirus, or transfected with the KRAS overexpression plasmid, or co-transfected with the miR-16-overexpressing lentivirus and the KRAS overexpression plasmid, and then the untreated or transfected cells were subcutaneously injected into C57BL/6J nude mice. After one month of xenograft growth *in vivo*, the mice were sacrificed and the weights of the tumors were measured. A marked reduction in the size and weight of the tumors was observed in the miR-16-overexpressing group compared to the control group, whereas the size and weight of the tumors in the KRAS-overexpressing group was dramatically increased. Additionally, KRAS overexpression attenuated the suppressive effect of miR-16 on tumor growth (Fig. 5A and B), suggesting that miR-16 may inhibit tumor growth by silencing KRAS. Next, total RNA and protein were isolated from the xenograft tumors and analyzed. Tumors from the miR-16-overexpressing group showed a significant increase in the expression of mature miR-16 compared to tumors from the control group (Fig. 5C). Likewise, KRAS mRNA levels were unchanged in tumors from the miR-16-overexpressing group but increased in tumors from the KRAS-overexpressing group (Fig. 5D). Moreover, tumors from the miR-16-overexpressing group displayed reduced KRAS protein levels compared to tumors from the control group, whereas tumors from the KRAS-overexpressing group showed elevated KRAS protein levels (Fig. 5E and F). More importantly, tumors with both miR-16 and KRAS overexpression exhibited significantly higher levels of KRAS than tumors with miR-16 overexpression (Fig. 5E and F), suggesting that KRAS overexpression could rescue the KRAS suppression caused by miR-16. Additionally, H&E staining of xenograft tissues showed less cell mitosis in the group implanted with the miR-16 lentivirus, whereas more cell mitosis was observed in the KRAS overexpression group (Fig. 5G). Xenografts with both miR-16 and KRAS overexpression exhibited more cell mitosis than xenografts with miR-16 overexpression (Fig. 5G). Immunohistochemical staining also revealed the presence of lower levels of KRAS in the tumors from mice implanted with miR-16-overexpressing cells, whereas the tumors from the KRAS-overexpressing mice showed increased KRAS protein levels (Fig. 5G and H). Finally, the proliferative activity of the tumor cells was assessed via immunocytochemistry with the mouse monoclonal antibody Ki-67. The cell proliferation rate, which was measured by the percentage of Ki-67-positive tumor cells, was decreased in the group implanted with the miR-16 lentivirus and increased in the group treated with the KRAS plasmid (Fig. 5G and I). Likewise, KRAS overexpression attenuated the anti-proliferative effect caused by miR-16 overexpression (Fig. 5G and I). These results were consistent with the findings of the *in vitro* assays, which firmly validated the tumor-suppressive role of miR-16 in CRC tumorigenesis through the targeting of KRAS.

## Discussion

CRC is one of the most common malignant human cancers worldwide. The development and progression of CRC is a complicated process that involves the dysregulation of a variety of genes that are essential for cellular processes^36^. Three members of the GTPases of the RAS family, KRAS, HRAS and NRAS, are well known for their ability to cause neoplasia. Among the RAS family, KRAS is one of the most prominent oncogenes due to its ability to transform human cells into malignant tumor cells, particularly when harboring an activating mutation in codon 12 or 13^37^. KRAS mutations frequently occur in many types of human cancers, for example in 70–90% of pancreatic cancer, 30–60% of colon cancer and 15–50% of lung cancer patients^12^. Moreover, activating oncogenic KRAS mutations are often associated with resistance to chemotherapy and targeted therapies in CRC^38^,^39^. Due to the poor prognosis for cancer patients with mutated KRAS, much effort has been spent on developing specific therapies for targeting oncogenic KRAS. However, technical limitations make it difficult to specifically inhibit KRAS *in vivo*. Apart from specific RNA interference methods, currently, there are no small molecules available that can specifically target KRAS. In this study, we showed that silencing KRAS expression could inhibit cell proliferation and invasion and induce apoptosis in CRC cells, while overexpressing KRAS had the opposite effects on CRC cells, validating the role of KRAS as a crucial oncogene during CRC tumorigenesis. More importantly, this study identified miR-16 as a novel link between the KRAS regulatory pathway and CRC and pointed the important role of miR-16 as a tumor suppressor in CRC through the inhibition of KRAS translation. Considering that miR-16 is an upstream regulator of KRAS, it is possible to restore miR-16 expression to inhibit KRAS expression *in vivo*. Currently, the overexpression of miRNAs can be silenced using antagomirs, and the re-expression of miRNAs that are lost in cancers can be achieved by the overexpression of miRNA mimics. Greater research emphasis is needed to characterize the feasibility of targeting miR-16 in CRC therapy and develop simplified and cost-effective manipulation methods.

miR-16 was first found to be associated with chronic lymphocytic leukemia^40^,^41^. Subsequent studies demonstrated that miR-16 is downregulated and serves as a tumor suppressor in a variety of cancer types. For example, Rivas *et al*. showed that downregulation of miR-16 via progestin-mediated oncogenic signaling contributed to breast cancer development^42^. Ma *et al*. reported that miR-16 could inhibit proliferation and induce the apoptosis of CRC cells by regulating the P53/survivin signaling pathway^43^. Ke *et al*. found that miR-16 was downregulated in non-small cell lung cancer cells and had an inverse correlation with the protein expression of hepatoma-derived growth factor^44^. Liang *et al*. reported that miR-16 showed an inverse correlation with the protein expression of FEAT in breast cancer, lung cancer and hepatocellular cancer tissues and that the overexpression of miR-16 promoted the apoptosis of cancer cells by targeting FEAT^45^. In this study, we identified a novel regulatory network that employs miR-16 and KRAS to regulate cell proliferation, invasion and apoptosis in CRC cells. We also provided evidence that the restoration of KRAS expression could partly rescue miR-16-suppressed cell proliferation and invasion and miR-16-induced apoptosis, suggesting that the targeting of KRAS is an important mechanism by which miR-16 exerts its tumor-suppressive function. Because the detailed roles that miR-16 plays in the initiation and progression of CRC remain not fully understood, the results of this study may explain, at least in part, why the downregulation of miR-16 during CRC carcinogenesis can promote cancer progression.

## Conclusions

In this study, we found that the expression levels of KRAS were significantly higher in CRC clinical tissues than in the non-tumor adjacent tissues. In addition, we showed for the first time that KRAS is a direct target of miR-16. We also provided evidence that miR-16 could inhibit the proliferation and invasion and induce the apoptosis of CRC cells by silencing KRAS. Furthermore, experiments using tumor xenografts in mice validated the tumor-suppressive role of miR-16 in CRC tumorigenesis through the targeting of KRAS. Taken together, this study highlights an important role for miR-16 in the regulation of KRAS in CRC cells and may open new avenues for future CRC therapy.

## Additional Information

**How to cite this article**: You, C. *et al*. Deregulation of the miR-16-KRAS axis promotes colorectal cancer. *Sci. Rep*. **6**, 37459; doi: 10.1038/srep37459 (2016).

**Publisher’s note:** Springer Nature remains neutral with regard to jurisdictional claims in published maps and institutional affiliations.

## Supplementary Material

Supplementary Information

## Figures and Tables

**Table 1:** Clinical features of CRC patients.

	Age	Gender	Tumor subtype	Pathological stage
Case #1	73	male	CRC	IIIB
Case #2	62	male	CRC	IIA
Case #3	64	female	CRC	IIIB
Case #4	71	male	CRC	IIIB
Case #5	65	female	CRC	IIIB
Case #6	60	male	CRC	IIIB
Case #7	68	male	CRC	IIIA
Case #8	64	female	CRC	IIB
Case #9	74	male	CRC	IIIA
Case #10	69	female	CRC	IIIB
Case #11	65	female	CRC	IIIA
Case #12	60	male	CRC	IIB
Case #13	72	male	CRC	IIIB
Case #14	63	female	CRC	IIIA
Case #15	70	male	CRC	IIIB
Case #16	62	male	CRC	IIA
