# Author Correction: Deregulation of the miR-16-KRAS axis promotes colorectal cancer

**DOI:** 10.1038/s41598-021-99119-w

**Published:** 2021-09-30

**Authors:** Chaoying You, Hongwei Liang, Wu Sun, Jialu Li, Yanqing Liu, Qian Fan, Haiyang Zhang, Xin Yue, Jing Li, Xi Chen, Yi Ba

**Affiliations:** 1 Tianjin Medical University Cancer Institute and Hospital, National Clinical Research Center for Cancer, Key Laboratory of Cancer Prevention and Therapy, Huanhuxi Road, Tiyuanbei, Tianjin, 300060 China; 2 State Key Laboratory of Pharmaceutical Biotechnology, NJU Advanced Institute for Life Sciences (NAILS), Jiangsu Engineering Research Center for MicroRNA Biology and Biotechnology, School of Life Sciences, Nanjing University, 163 Xianlin Avenue, Nanjing, 210046 China; 3 Department of Gastroenterology, Tianjin First Center Hospital, 24 Fukang Road, Tianjin, 300192 China

Correction to: *Scientific Reports* https://doi.org/10.1038/srep37459, published online 18 November 2016

The original version of this Article contains errors.

In Figure 4D, the representative images for the cell invasion assay experiments “pre-miR-control+control vector” and “pre-miR-16+KRAS vector” are partially duplicated from the image showing “pre-miR-control”.

The correct Figure 4 and accompanying legend appear below.

**Figure 4:**
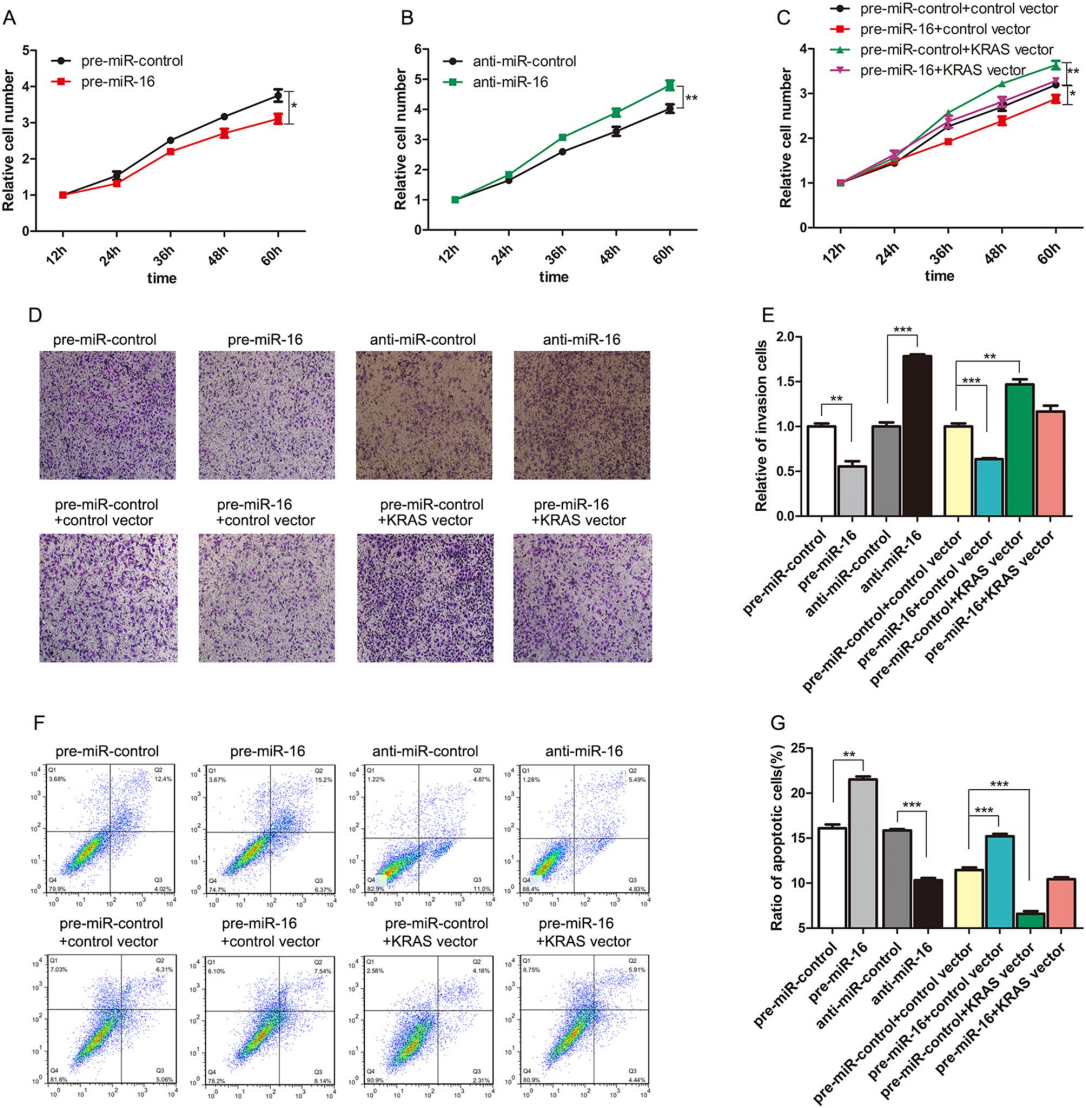
Effect of miR-16 and KRAS on the proliferation, invasion and apoptosis of CRC cells. (**A**) Cell proliferation assays were performed 12, 24, 36, 48 and 60 h after the transfection of SW480 cells with pre-miR-16 or pre-miR-control. (**B**) Cell proliferation assays were performed 12, 24, 36, 48 and 60 h after the transfection of Caco2 cells with anti-miR-16 or anti-miR-control. (**C**) Cell proliferation assays were performed 12, 24, 36, 48 and 60 h after the transfection of SW480 cells with pre-miR-control plus a control plasmid, pre-miR-control plus a KRAS overexpression plasmid, pre-miR-16 plus a control plasmid, or pre-miR-16 plus a KRAS overexpression plasmid. (**D** and **E**) Transwell analysis of SW480 cells transfected with pre-miR-16 or pre-miR-control, or with pre-miR-control plus a control plasmid, pre-miR-control plus a KRAS overexpression plasmid, pre-miR-16 plus a control plasmid, or pre-miR-16 plus a KRAS overexpression plasmid. At the same time, Caco2 cells were transfected with anti-miR-16 or anti-miR-control and then subjected to Transwell analysis. D: representative image; E: quantitative analysis. (**F** and **G**) An apoptosis assay was performed 48 h after the transfection of SW480 cells with pre-miR-16 or pre-miR-control, or with pre-miR-control plus a control plasmid, pre-miR-control plus a KRAS overexpression plasmid, pre-miR-16 plus a control plasmid, or pre-miR-16 plus a KRAS overexpression plasmid. At the same time, Caco2 cells were transfected with anti-miR-16 or anti-miR-control and then subjected to apoptosis analysis. Cell apoptosis profiles were analyzed by flow cytometry. F: representative image; G: quantitative analysis. (mean ± S.D.; *p < 0.05; **p < 0.01; ***p < 0.001).

