# Supplementary material for: Deregulation of the miR-16-KRAS axis promotes colorectal cancer
Source: Sci Rep. 2016 Nov 18;6:37459. doi: 10.1038/srep37459 (PMC5114589; doi:10.1038/srep37459)

**Supplementary Materials**

**Deregulation of the miR-16-KRAS axis promotes colorectal cancer**

Chaoying You, Hongwei Liang, Wu Sun, Jialu Li, Yanqing Liu, Qian Fan, Haiyang Zhang, Xin Yue, Jing Li, Xi Chen, Yi Ba

**Supplementary Figure 1. The mean expression levels of KRAS protein and mRNA and miR-16 in the CRC and NAT samples. (A)** The mean expression levels of KRAS protein in 16 pairs of CRC and NAT samples. **(B)** The mean expression levels of KRAS mRNA in 16 pairs of CRC and NAT samples. **(C)** The mean expression levels of miR-16 in 16 pairs of CRC and NAT samples. (mean ± S.D.; *** p < 0.001).

**
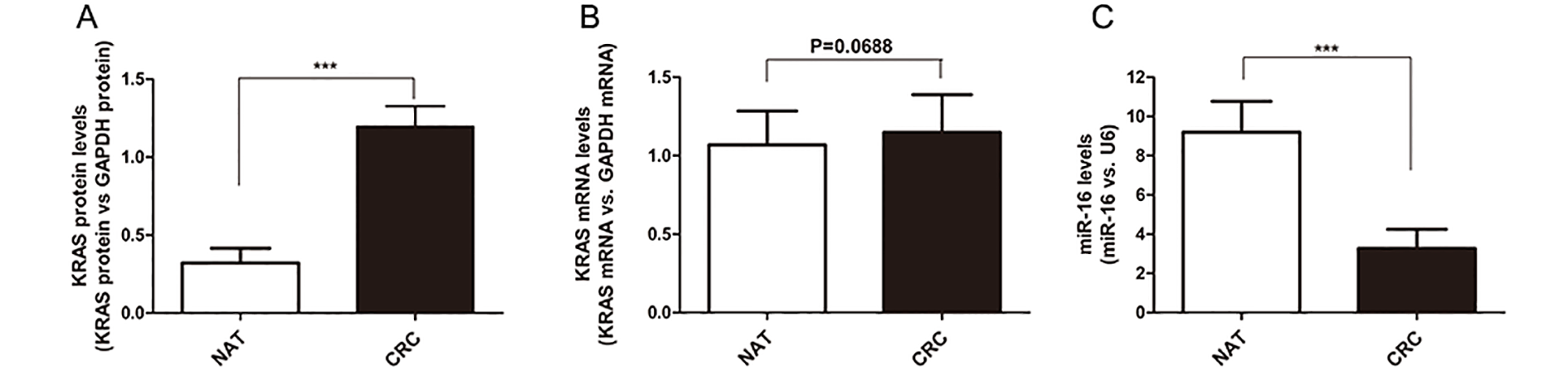
**

**Supplementary Figure 2. Effect of KRAS on the proliferation, invasion and apoptosis of CRC cells. (A)** Quantitative RT-PCR analysis of the KRAS mRNA levels in SW480 cells transfected with control siRNA, KRAS siRNAs (#1-#4), control plasmid or KRAS overexpressing plasmid. **(B and C)** Western blot analysis of the KRAS protein levels in SW480 cells transfected with control siRNA, KRAS siRNAs (#1-#4), control plasmid or KRAS overexpressing plasmid. B: representative image; C: quantitative analysis. **(D)** Cell proliferation assays were performed 12, 24, 36, 48 and 60 h after the transfection of SW480 cells with control siRNA or KRAS siRNA. **(E)** Cell proliferation assays were performed 12, 24, 36, 48 and 60 h after the transfection of SW480 cells with control plasmid or KRAS overexpressing plasmid. **(F and G)** Transwell analysis of SW480 cells treated with equal doses of control siRNA, KRAS siRNA, control plasmid, or KRAS overexpressing plasmid. F: representative image; G: quantitative analysis. **(H and I)** Cell apoptosis profiles were analyzed by flow cytometry in SW480 cells transfected with equal doses of control siRNA, KRAS siRNA, control plasmid, or KRAS overexpressing plasmid. H: representative image; I: quantitative analysis. (mean ± S.D.; ** p < 0.01; *** p < 0.001)

**
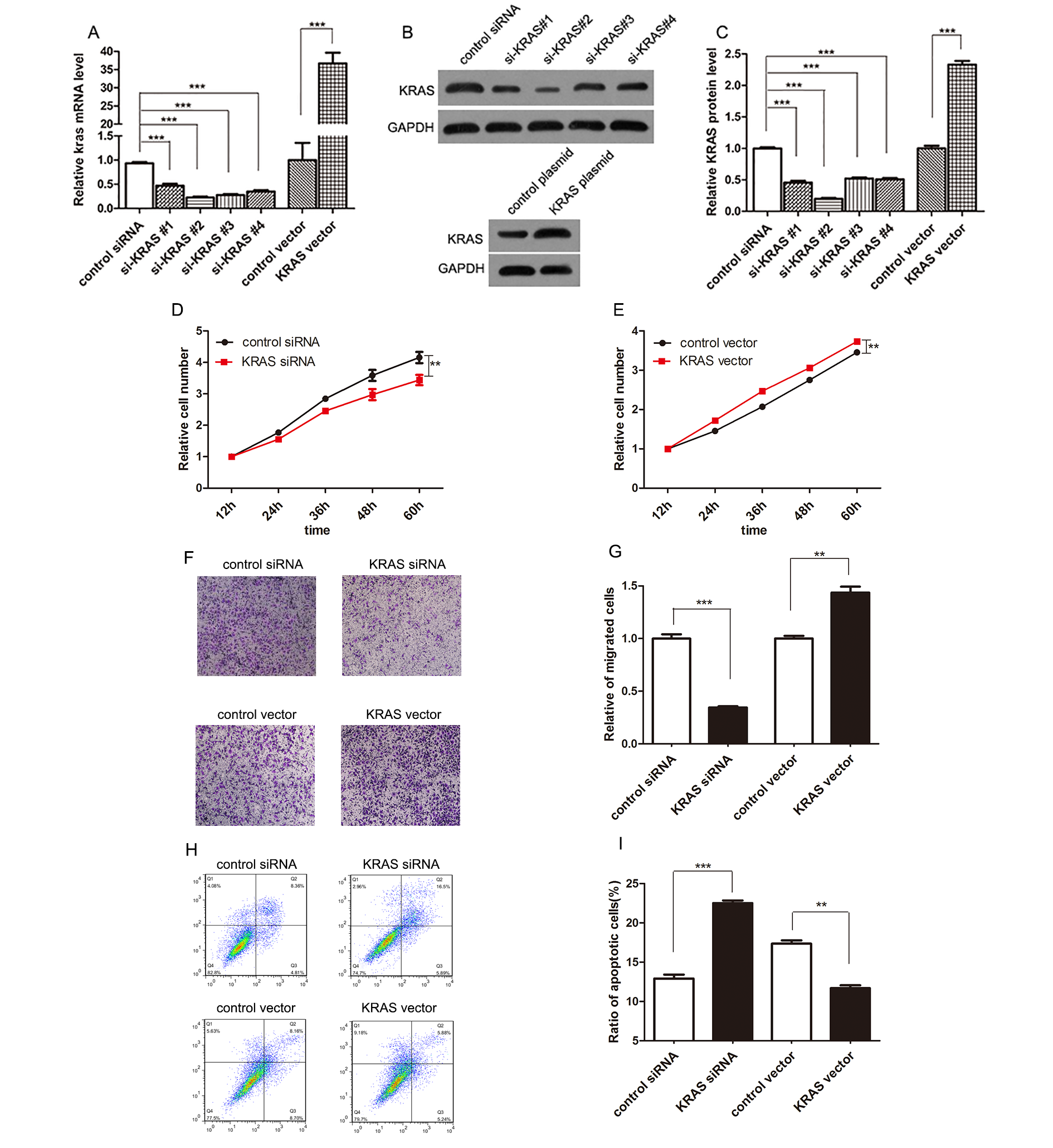
**

**Supplementary Figure 3. Expression levels of miR-16 and KRAS in SW480 cells after transfection with a lentivirus to overexpress miR-16. (A)** Quantitative RT-PCR analysis of the miR-16 levels in mock-treated SW480 cells and SW480 cells transfected with a lentivirus to overexpress miR-16. **(B and C)** Western blotting analysis of the KRAS protein levels in mock-treated SW480 cells and SW480 cells transfected with a lentivirus to overexpress miR-16. B: representative image; C: quantitative analysis. (mean ± S.D.; *** p < 0.001).


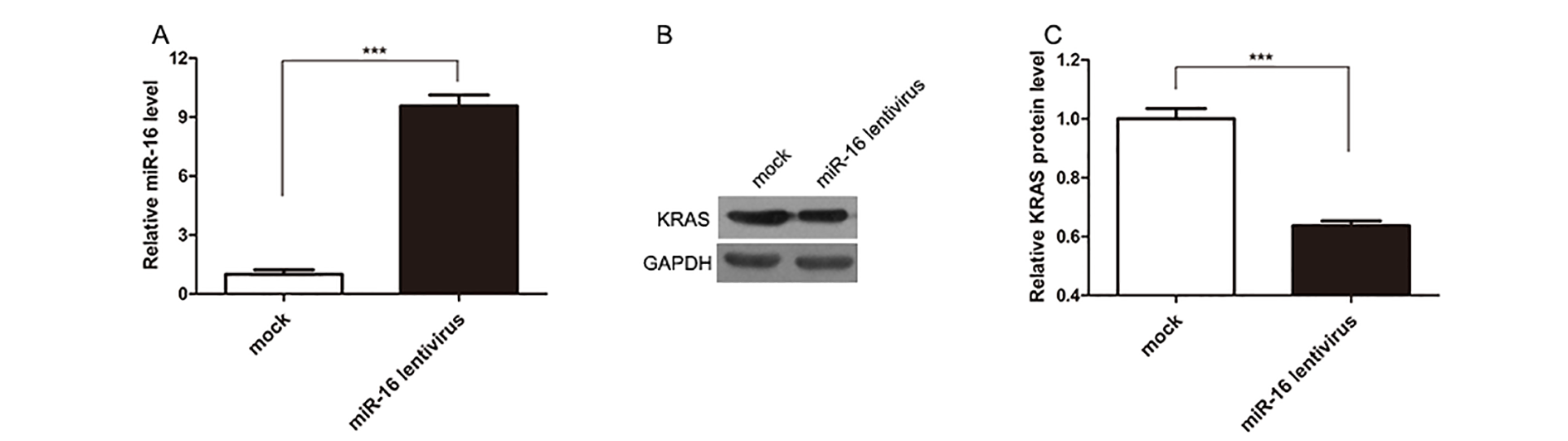


**Supplementary Figure 4. The parent unmodified Western blot images without cropping. (A)** Western blot analysis of the KRAS protein levels in 16 pairs of CRC and NAT samples. GAPDH was used as a loading control (N: NAT; C: CRC). **(B)** Western blot analysis of the KRAS protein levels in SW480 and HT-29 cells that were transfected with pre-miR-16 or pre-miR-control and in Caco2 cells that were transfected with anti-miR-16 or anti-miR-control for 48 h. **(C)** Western blot analysis of the KRAS protein levels in SW480 cells that were transfected with control siRNA, KRAS siRNAs (#1-#4), control plasmid or KRAS overexpressing plasmid. **(D)** Western blotting analysis of the KRAS protein levels in mock-treated SW480 cells and SW480 cells transfected with a lentivirus to overexpress miR-16; Western blotting analysis of the KRAS protein levels in tumors from the implanted mice.


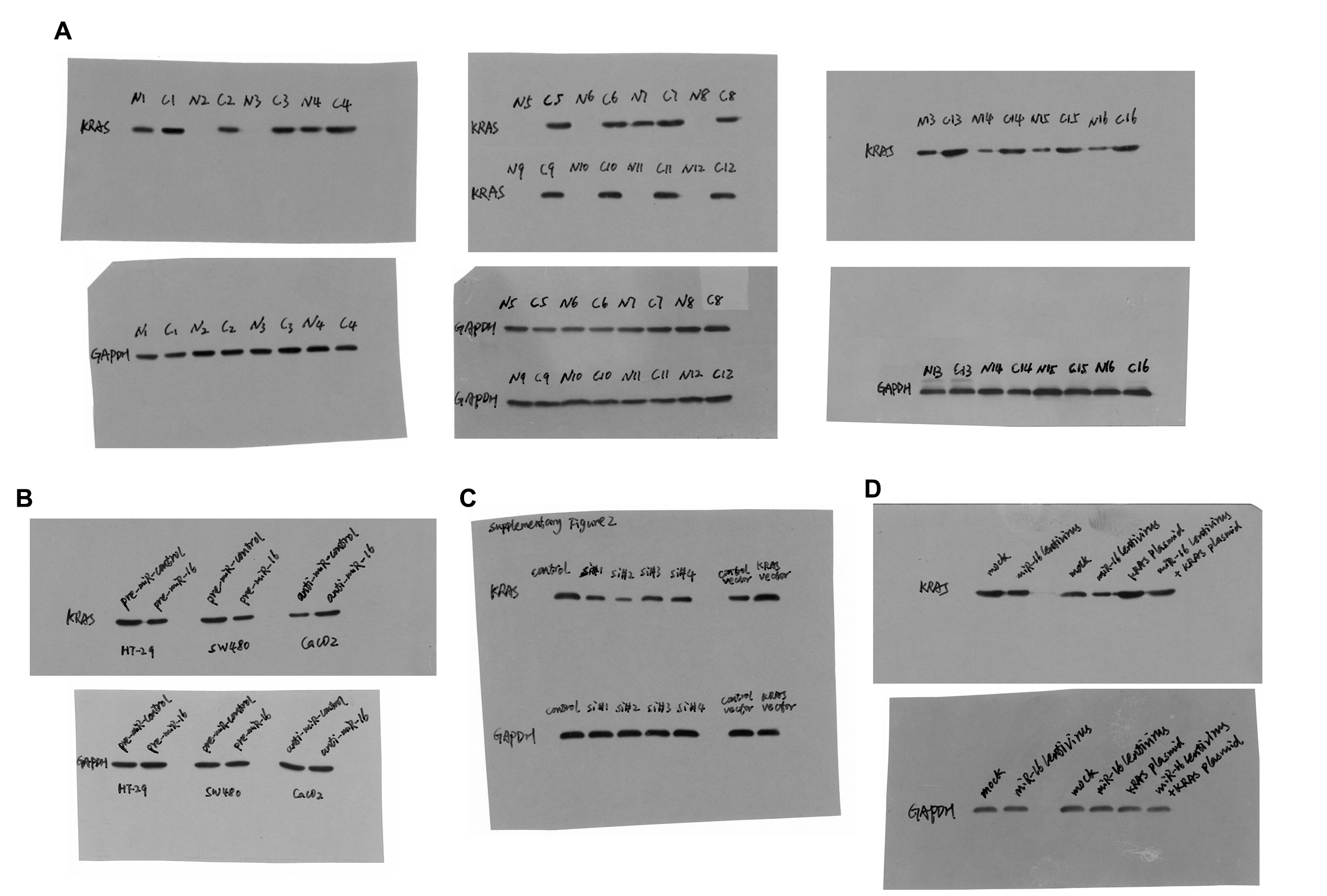

Supplement: Supplementary Information [file srep37459-s1.doc]
